# Using inactivating mutations to provide insight into drug action

**DOI:** 10.1186/s13073-015-0130-x

**Published:** 2015-01-28

**Authors:** Laura J Corbin, Nicholas J Timpson

**Affiliations:** MRC Integrative Epidemiology Unit (IEU), University of Bristol, Oakfield House, Oakfield Grove, Bristol, BS8 2BN UK

## Abstract

The role of ezetimibe in lowering plasma cholesterol has been established; however, controversy remains about its clinical benefit. A recent study utilizes naturally occurring genetic variation within the NPC1-like 1 gene (*NPC1L1*) to demonstrate the potential for pharmacologic inhibition of the protein to reduce the risk of coronary heart disease. This research demonstrates the application of the concept of genocopy to a population-based validation of *NPC1L1* as a therapeutic target.

## Ezetimibe as an adjunct to statin therapy in cardiovascular disease

Cardiovascular diseases (CVDs) are the primary cause of death globally [1]. In 2008, 30% of all global deaths were attributed to CVDs, including an estimated 7.3 million deaths caused by coronary heart disease (CHD) [1]. As a major risk factor for CHD, increased circulating cholesterol - particularly low-density lipoprotein cholesterol (LDL-C) - is a well-established target for clinical intervention. Although treatment with 3-hydroxy-3-methyl-glutaryl-CoA reductase (HMGCR) inhibitors (statins) has been shown both to lower LDL-C levels and to reduce major cardiovascular events, in some cases statin therapy alone is insufficient to achieve optimal LDL-C levels [2]. Currently, ezetimibe, which inhibits the function of the NPC1L1 protein, can be prescribed alongside statins in order to achieve further reductions in LDL-C or as an alternative in instances where statins are contraindicated. Whilst the ability of ezetimibe to independently and additively lower LDL-C beyond the levels achieved by statins alone does not appear to be in question, the degree to which the drug contributes to a reduction in the risk of clinically relevant cardiovascular outcomes such as CHD is unclear.

Numerous trials have been conducted to assess the clinical utility of LDL-C-lowering therapies in reducing the incidence of CVD. The Pravastatin or Atorvastatin Evaluation and Infection Therapy Trial (PROVE-IT) demonstrated that more intensive lipid lowering achieved through an increased statin dose clinically benefited patients who had previously suffered an acute coronary syndrome [3]. However, whether the same benefits can be achieved by prescribing ezetimibe alongside statins to achieve similar reductions in LDL-C remains uncertain owing to inconsistent trial outcomes, particularly where endpoints such as carotid intimal thickening and vascular reactivity have been used as surrogates for CVD risk [2].

Two of the largest randomized control trials (RCTs) designed to determine whether adding ezetimibe to statins provides clinical benefit (over and above statin monotherapy) carried out to date are the Ezetimibe and Simvastatin in Hypercholesterolemia Enhances Atherosclerosis Regression (ENHANCE) trial and IMProved Reduction of Outcomes: Vytorin Efficacy International Trial (IMPROVE-IT). The ENHANCE trial (in which patients with heterozygous familial hypercholesterolemia received simvastatin with or without ezetimibe) was designed to study the effect of ezetimibe on the progression of atherosclerosis, using carotid intima-media thickness (IMT) as a target endpoint. In this trial, the addition of ezetimibe to simvastatin therapy in the treatment of familial hypercholesterolemia did not produce a reduction in carotid IMT, despite achieving a differential reduction in LDL-C [4]. This result was contrary to findings of similar trials conducted around the same time [2]. In November 2014, preliminary results of the landmark IMPROVE-IT, designed to determine whether adding ezetimibe to simvastatin in patients presenting with acute coronary syndromes adds clinical benefit by further reducing major cardiovascular events compared with simvastatin monotherapy [5], were presented at the American Heart Association Annual Meeting (Chicago, IL, Nov. 15-19, 2014). Although a full description of the findings is yet to be published, preliminary results apparently suggest a modest benefit in reducing cardiovascular events with the addition of ezetimibe to simvastatin in this population of around 18,000 patients from 39 countries. However, overall, the discordant results of studies carried out to date has generated a degree of scepticism about whether ezetimibe offers any health benefits above and beyond those afforded by statin therapy.

Using a population-based approach theoretically derived from the concept of genocopy, Stitziel *et al.* [6] offer evidence to strengthen inference from existing observational and RCT studies and to inform future research. The term ‘genocopy’ refers to genetic variation that generates an outcome similar to that produced by an environmental exposure [7]. An illustrative example of this phenomenon is the autosomal recessive condition of Hartnup disease. This disease is caused by a mutation in solute carrier family 6 member 19 (*SLC6A19*; the ‘genocopy’) but a very similar clinical manifestation occurs in cases of dietary niacin deficiency, a condition known as pellagra (the ‘phenocopy’). Using a Mendelian randomization approach that takes advantage of the properties of genetic variation and follows similar logic (such that mutations in *NCP1L1* act as genocopies mimicking the action of ezetimibe), Stitziel *et al.* [6] attempt to separate causation from association, providing validation of *NCP1L1* as a therapeutic target.

## Using human genetics to validate the role of *NCP1L1* in coronary heart disease

Using sequence data from over 22,000 individuals of varying ancestry, Stitziel and colleagues [6] identify 15 mutations that are predicted to inactivate *NCP1L1*. The mutations lie within one of the gene’s 20 protein-coding exons and, for the researchers to consider them as inactivating, they had to be classified as nonsense, splice-site or frameshift mutations. The variants identified are rare, being found in only around 1 in 650 participants, and existing only in a heterozygous state. The most frequently observed mutation was p.Arg406X, which had a minor allele frequency of 0.02% among participants of European ancestry. Stitziel and co-workers [6] went on to genotype this variant in a further nine independent sample sets, totalling 91,002 participants. This targeted genotyping afforded the study a considerable gain in statistical power, increasing the number of participants carrying an inactivating variant from 34 in 22,092 to 82 in 113,094.

Data obtained for all the participants in the study included medical history and laboratory assessments for cardiovascular risk factors. These data were combined with the genetic data, firstly to test the association between *NCP1L1* protein-activating mutations and plasma lipid levels, and subsequently to test for an association between these same mutations and risk of CHD. Association analyses revealed that carriers of any of the *NCP1L1* inactivating mutations identified had lower levels of total cholesterol, LDL-C and triglycerides. In an analysis that was restricted to participants characterized as being free from CHD, the mean LDL-C level was 12 mg per decilitre lower in carriers of an inactivating mutation than in non-carriers, while no difference was observed in high-density lipoprotein cholesterol.

Having used the clinical data available for each study to define CHD status, Stitziel *et al.* [6] went on to demonstrate an odds ratio for the disease among carriers of inactivating mutations of 0.47 (95% confidence interval, 0.25 to 0.87). The odds ratio was calculated based on carrier frequencies of 0.04% in 29,954 patients with CHD compared with frequencies of 0.09% in 83,140 unaffected participants. The apparent protective effect of *NCP1L1* protein-inactivating mutations against high plasma lipid levels and CHD observed in the entire cohort was also evident when the analysis was conducted separately in European and African subgroups.

## Clinical interpretation

By using naturally occurring genetic variants, Stitziel and colleagues [6] exploit a natural experiment that simulates the effect of exposure to ezetimibe and avoids many of the pitfalls associated with traditional observational studies. Of course, when interpreting the results of such studies it is important to take into consideration (as Stitziel and co-workers [6] do) that genetic models, such as this one for *NCP1L1* inactivation, are not a perfect proxy for pharmacologic therapy. For example, one important caveat is that the drug could have off-target effects that may not be modelled in the case of a single-gene framework. However, when it comes to modelling the long-term effects of treatment, such Mendelian randomization-style approaches are well placed to detect life course effects. Genetic variation can provide a natural life course model because it represents lifetime exposure to the effects of mutation, which in this case mirrors the action of ezetimibe [8,9].

The use of genetic variation to assess drug effects is an established approach and the identification of novel rare variants of large effect adds to its potential. Whether the use of such variants provides benefits over and above those seen using common variants is unclear, but it is possible that the resultant genetic model is one that more closely resembles the drug effect being simulated and has greater power to detect differences in disease risk because of the relatively large effect sizes observed. Whether or not this is the case will likely become clearer as more studies are published, with some insight already having been provided in a similar study in which multiple mutations in *NCP1L1* and *HMGCR* were considered in concert using a genetic score approach to explore the impact of combination therapy with statins and ezetimibe on CHD risk [10]. There are many situations in which the approach taken by Stitziel *et al.* [6] could be utilized, but one particularly important application might be in providing validation of proposed therapeutic targets before the clinical trial stage.

## Abbreviations

CHD: Coronary heart disease
CVD: Cardiovascular disease
IMPROVE-IT: IMProved Reduction of Outcomes: Vytorin Efficacy International Trial
IMT: Intima-media thickness
LDL-C: Low-density lipoprotein cholesterol
PROVE-IT: Pravastatin or Atorvastatin Evaluation and Infection Therapy Trial
RCT: Randomized control trial
